# Dopamine genetic risk score predicts impulse control behaviors in Parkinson’s disease

**DOI:** 10.1016/j.prdoa.2021.100113

**Published:** 2021-10-29

**Authors:** Alison Hall, Samuel R. Weaver, Lindsey J. Compton, Winston D. Byblow, Ned Jenkinson, Hayley J. MacDonald

**Affiliations:** a School of Sport, Exercise and Rehabilitation Sciences, University of Birmingham, Birmingham, UK; b Centre for Human Brain Health, University of Birmingham, Birmingham, UK; c School of Biosciences, University of Birmingham, Birmingham, UK; d Department of Exercise Sciences, University of Auckland, Auckland, New Zealand; e Centre for Brain Research, University of Auckland, Auckland, New Zealand

**Keywords:** Parkinson’s disease, Impulse control behavior, Dopamine agonist, Parkinson’s Progression Markers Initiative

## Abstract

**Introduction:**

Up to 40% of Parkinson’s disease patients taking dopamine agonist medication develop impulse control behaviors which can have severe negative consequences. The current study aimed to utilize dopamine genetics to identify patients most at risk of developing these behaviors.

**Methods:**

Demographic, clinical, and genetic data were obtained from the Parkinson’s Progression Markers Initiative for de novo patients (n = 327), patients taking dopamine agonists (n = 146), and healthy controls (n = 160). Impulsive behaviors were identified using the Questionnaire for Impulsive-Compulsive Disorders in Parkinson’s Disease. A dopamine genetic risk score was calculated for each patient according to polymorphisms in genes coding for dopamine D1, D2 and D3 receptors, and catechol-O-methyltransferase. A higher score reflected higher central dopamine neurotransmission.

**Results:**

Patients on agonists with a low dopamine genetic risk score were over 18 times more likely to have an impulsive behavior compared to higher scores (p = 0.04). The 38% of patients taking agonists who had at least one impulsive behavior were more likely to be male and report higher Unified Parkinson’s Disease Rating Scale I&II scores. With increasing time on dopamine agonists (range 92–2283 days, mean 798 ± 565 standard deviation), only patients with a high dopamine genetic risk score showed an increase in number of impulsive behaviors (p = 0.033). Predictive effects of the gene score were not present in de novo or healthy control.

**Conclusions:**

A dopamine genetic risk score can identify patients most at risk of developing impulsive behaviors on dopamine agonist medication and predict how these behaviors may worsen over time.

## Highlights

• Dopamine gene score predicts impulsivity in Parkinson’s patients on agonists.
• A low gene score predicted an increased likelihood of impulse control problems.
• Gene score was associated with change in impulsivity over time on medication.

## 1 Introduction

Impulse control is an essential aspect of self-restraint. Dopamine systems are important regulators of impulse control, such that abnormal levels of dopamine can lead to problems with impulsivity [1], [2]. A significant risk factor for problems with impulse control in Parkinson’s disease (PD) patients is dopamine agonist (DA) medication. Up to 40% of patients administered DAs develop impulse control disorders (ICDs) e.g., pathological gambling, binge eating, compulsive shopping or hypersexuality [3], [4], [5]. Here, we use the term impulse control behaviors (ICBs), a term commonly used to describe all ICDs and related behaviors [6].

Sparing of specific dopaminergic networks in PD combined with dopamine medication may elevate the risk of ICBs. In early, unmedicated (de novo) PD there is a reduction of nigrostriatal dopamine [1], [7]. In contrast, dopamine is relatively spared in the amygdala, ventral striatum, prefrontal cortex and orbital frontostriatal circuits, constituting the mesocorticolimbic (MCL) system [8], [9], [10]. The MCL system is heavily involved in impulse control [1]. Furthermore, in PD there is heightened sensitivity of D2/D3 receptors following dopamine denervation from the midbrain to areas of the ventral striatum, which in turn further increases dopamine activity in the connecting MCL regions [11], [12]. However, dysregulation of MCL dopamine from PD mechanisms alone is insufficient to increase the incidence of ICBs in de novo PD compared to controls [13], [14], [15]. The addition of DA medication is thought to cause a dopamine overdose within the relatively spared MCL system [8], [16]. DA medications primarily act on D2/D3 receptors [17], further increasing their activity. The resultant tonic hyperdopaminergic state can lead to problems with phasic dopamine modulation and over time the development of ICBs [1], [2], [7].

Using genetics to guide precision medicine is fast gaining traction and may be applicable to reduce the incidence of ICB side-effects. Polymorphisms within single dopamine genes are individually associated with the incidence of ICBs in PD [18], [19], [4], [5], [6], though no study to date has investigated the collective influence of multiple genetic polymorphisms as a genetic risk score on widespread central dopamine levels and ICBs. A polygenic dopamine genetic risk score (DGRS) [20], [21], [22] is a strong candidate for quantifying widespread tonic dopamine neurotransmission. A DGRS quantifies the effect of polymorphisms within five key genes that modify dopamine neurotransmission within MCL regions [9], [10], [12] and affect impulse control [5], [6], [9], [12], [18], [23]: DRD1, DRD2, DRD3 (encoding D1, D2 and D3 receptors, respectively), catechol-O-methyltransferase (COMT), and dopamine transporter (DAT). A DGRS can predict impulse control in healthy older adults, including how impulse control will change with administration of DAAAs [22]. In that study [22], both motor and cognitive aspects of impulse control were worse for participants with a low versus high DGRS at baseline. Ropinirole caused worsening impulse control for participants with a high DGRS, whereas participants with a low DGRS saw improvements.

The present study investigated whether a DGRS can predict ICBs in a large sample of PD patients. Demographic, clinical and genetic data were obtained from the Parkinson’s Progression Markers Initiative (PPMI) database. The primary aim was to determine the association between DGRS and the development of ICBs, within de novo and PD patients on DAs. The secondary aim was to establish which demographic and clinical variables were associated with ICBs and whether they interacted with the DGRS. We hypothesized that patients on DAs with a low DGRS would be more likely to have an ICB, but that ICBs would reduce over time on medication. Conversely, we hypothesized that patients with a high DGRS would be less likely to have an ICB, but that ICBs would increase with greater time on medication. We further hypothesized that ICBs would be associated with male gender and a higher Unified Parkinson’s Disease Rating Scale (UPDRS) I&II score [4], [19], [24]. Genetic and demographic data from healthy controls were included and no associations were expected with ICBs.

## 2 Materials and methods

### 2.1 Participants

The PPMI is an ongoing, cohort database including demographic, clinical, imaging, genetic and biological data for PD patients and healthy controls. PPMI is a public–private partnership, funded by the Michael J. Fox Foundation for Parkinson’s Research and funding partners (http://www.ppmi-info.org). Clinical and demographic data from 2035 individuals were downloaded on 22 October 2018 and genetic data on 29 April 2019. Individuals were categorized into three groups: de novo (DN): with PD before medication, dopamine agonist (DA): with PD taking DA medication, or healthy control (HC).

### 2.2 Clinical measures

Impulse control was measured via the short form of the Questionnaire for Impulsive-Compulsive Disorders in Parkinson’s Disease (QUIP-short), a globally validated screening tool to identify ICBs with any positive score [25]. The QUIP involved answering ‘yes’ or ‘no’ to 13 questions, resulting in a score of 1 or 0 for each question, respectively. Total scores therefore ranged from 0 to 13. QUIP score and current age were taken at maximum time since starting DAs/PD diagnosis/study enrolment, as appropriate. Duration refers to continuous time on DA medication/time since diagnosis at the time of the QUIP. Severity of PD symptoms in activities of daily living was assessed using the UPDRS parts I&II [26]. UPDRS parts III and IV were not available from a sufficient number of patients to include in the study.

### 2.3 Genetic data

Five specific genetic polymorphisms were identified for analysis *a priori* [22]. However, data was not available to analyze the variable number tandem repeat in the DAT gene (rs28363170) as the untranslated regulatory region of this gene was not genotyped. Exome sequencing files for the remaining four single nucleotide polymorphisms (SNPs) were used. Exome sequencing was performed on whole-blood extracted DNA samples using an Illumina rapid capture expanded exome kit. Sequencing data was aligned against the University of California Santa Cruz reference human genome 19 to find the 4 genotype locations for each SNP using GATK (VariantsToTable, version 4.1.2.0). For complete methods, see Exome Sequencing Methods (project 116), (http://www.ppmi-info.org/data). The DGRS (Table 1S, supplemental material) was adapted to a scale of 0–4 (higher score = higher dopamine levels) according to the SNP within the following genes: DRD1 (rs4532), DRD2 (rs1800497), DRD3 (rs6280) and COMT (rs4680) [20], [21]. All genes apart from COMT (p = 0.008) were in Hardy-Weinberg equilibrium (0.07 > p < 0.86).

### 2.4 Statistical analysis

Data analysis and statistical modelling were performed in MATLAB (version R2020a, MathWorks) and R (R Core Team, version 3.6.3). Chi-square tests assessed Hardy–Weinberg equilibrium for each gene. Normality assumptions were checked using the Kolmogorov-Smirnov test. When normality was violated, data were analyzed using the Wilcoxon Rank-Sum test. Statistical significance was set at p ≤ 0.05.

#### 2.4.1 ICB incidence

**Fig. 1:**
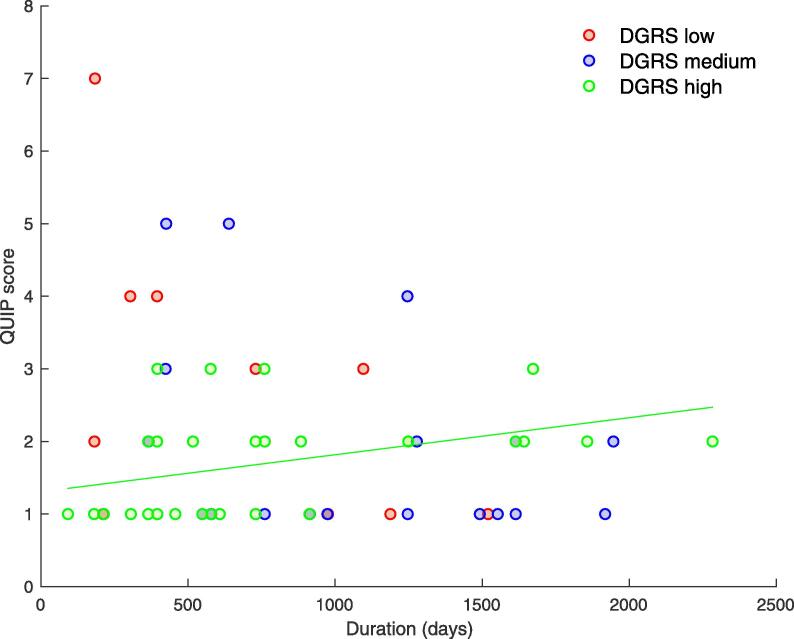
Change in score on Questionnaire for Impulsive-Compulsive Disorders in Parkinson’s Disease (QUIP) over time on agonist medication, categorized by dopamine genetic risk score (DGRS).

**Fig. 2:**
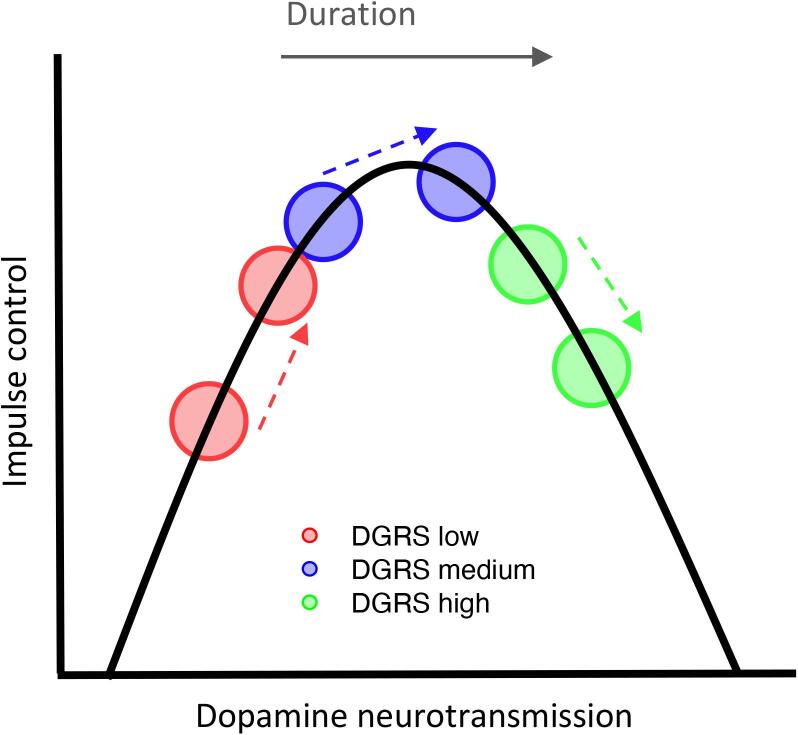
Inverted-U relationship between dopamine neurotransmission and impulse control. Increased time on agonist medication (dashed arrows) moves individuals rightwards along the curve. Changes to impulse control depend on dopamine genetic risk score (DGRS) i.e. starting point on curve.

ICB incidence was defined as any positive score on the QUIP. Candidate independent variables were age, DGRS, duration, gender and UPDRS I&II score [3], [4], [22], [27], [28]. The DGRS was categorized into three ranges: low (DGRS 0–1), medium (DGRS 2, reference variable in regression analyses) and high (DGRS 3–4) to increase sample size for each group. Linear regressions were run between continuous variables to test for collinearity. If collinearity existed, variables were removed from the model to avoid overparameterization. The relationship between each independent variable and the response variable was initially investigated using univariate binary logistic regression analyses (see supplemental material), which confirmed variables to include in the full model. A multivariate binary logistic model was developed which included the selected independent variables and important interactions. Model validation (i.e. goodness-of-fit) was assessed against a constant model using a chi-squared test (p < 0.05). Two receiver operating characteristic (ROC) curves were produced for each participant group’s multivariate model to evaluate specific changes to the predictability of incident ICB following the inclusion of the DGRS (Fig. 1S & 2S). Resultant AUC values were compared using DeLong’s test [29].

#### 2.4.2 QUIP score and medication

Correlations between QUIP score (i.e. number of ICBs) and time on DA medication were run for each DGRS range (low, medium, high). Fisher z transformations identified differences between correlations.

## 3 Results

### 3.1 Participant characteristics

Data from 506 individuals (36–89 years, mean 63.7 ± 9.92 standard deviation) were included in the analysis (DN = 327; DA = 146; HC = 160; 127 DA patients had data since de novo stage so contributed to both DN and DA groups). Patients had a DGRS of low, medium or high. The number of patients with each DGRS for every group was as follows: DN group Low: n = 44, Medium: n = 106, High: n = 177; DA group Low: n = 23, Medium: n = 50, High: n = 73; HC Low: n = 24, Medium: n = 45, High: n = 91.

Demographic and clinical data are presented in Table 1. Kolmogorov-Smirnov tests identified the QUIP score in all groups and the UPDRS I&II score in the DA group violated normality (p < 0.001), therefore a Wilcoxon rank sum test was used to compare scores between individuals with/without ICBs. All remaining variables were normally distributed (p > 0.194) so comparisons were made using unpaired t-tests. Patients in the DN group who identified an ICB had a higher UPDRS score (p = 0.007) than those without an ICB. In the DA group, a greater number of males (p = 0.041) and patients with a higher UPDRS (p < 0.001) presented with an ICB. In the HC group, there was no difference in variables between those with and without an ICB (all p > 0.383).

**Table 1:** Participant demographics and clinical assessments for de novo, dopamine agonist and healthy control groups, separated by incidence of impulse control behaviours.

De novo (DN)			
	ICB (n = 43)	No ICB (n = 284)	p
Age, years	61.3 (9.45)	62.7 (9.83)	0.366
DGRS 0–4	2.39 (1.05)	2.54 (0.95)	0.105
Duration, days	497 (316)	530 (357)	0.569
Gender, %male (n, male:female)	58.1 (25:18)	67.6 (192:92)	0.222
**QUIP score**	**1.58 (0.76)**	**0**	**<0.001♦**
**UPDRS I&II**	**18.2 (9.73)**	**14.5 (8.13)**	**0.007**

**Dopamine agonist (DA)**			

	**ICB (n = 56)**	**No ICB (n = 90)**	**p**

Age, years	62.8 (9.35)	63.3 (7.61)	0.712
DGRS 0–4	2.39 (0.93)	2.44 (1.03)	0.760
Duration, days	869 (554)	843 (567)	0.785
**Gender, %male (n, male:female)**	**71.4 (40:16)**	**54.4 (49:41)**	**0.041**
**QUIP score**	**1.96 (1.26)**	**0**	**<0.001♦**
**UPDRS I&II**	**23.8 (12.2)**	**16.4 (11.0)**	**<0.001♦**

**Healthy control (HC)**			

	**ICB (n = 25)**	**No ICB (n = 135)**	**p**

Age, years	65.5 (12.7)	66.9 (10.7)	0.570
DGRS	2.48 (0.96)	2.54 (0.92)	0.764
Gender, %male (n, male:female)	56.0 (14:11)	65.2 (88:47)	0.383
**QUIP score**	**1.52 (0.71)**	**0**	**<0.001♦**

Means for variables (±standard deviation). ICB: impulse control behaviour (n: number). DGRS: dopamine genetic risk score; QUIP: Questionnaire for impulsive-Compulsive Disorders in Parkinson’s Disease; UPDRS: Unified Parkinson’s Disease Rating Scale. Significant values in bold. ♦: Wilcoxon rank sum test.

### 3.2 ICB incidence

#### 3.2.1 Dopamine agonist group

DGRS, duration, gender and UPDRS I&II score were included in the model with DGRS × duration and DGRS × UPDRS I&II interactions (Table 2). Age was excluded to avoid over-parameterization following univariate analysis (Table 2S).

**Table 2:** Variables associated with impulse control behaviours in the dopamine agonist group.

	β	SE	p value	Odds/OR
**Intercept**	**−3.819**	**1.141**	**<0.001**	**0.02**
**DGRS low**	**2.896**	**1.417**	**0.04**	**18.1**
DGRS high	1.851	1.274	0.146	6.40
Duration (days)	0.0007	0.0006	0.206	1.00
**Gender (male)**	**0.817**	**0.405**	**0.044**	**2.26**
**UPDRS I&II**	**0.088**	**0.034**	**0.01**	**1.09**
DGRS low * Duration	−0.001	0.001	0.161	1.00
DGRS high * Duration	−0.0007	0.0008	0.257	1.00
DGRS low * UPDRS I&II	−0.048	0.05	0.338	0.95
DGRS high * UPDRS I&II	−0.032	0.042	0.455	0.97

Response variable: positive score on Questionnaire for Impulsive-Compulsive Disorders in Parkinson’s Disease (yes/no). DGRS: dopamine genetic risk score, UPDRS: Unified Parkinson’s Disease Rating Scale. β: coefficient, SE: standard error, OR: odds ratio (OR = e^β^). Significant values in bold.

Binary logistic regression function:

$$p=\frac{{exp(\beta }_{0}\left(intercept\right)+\beta_{1}DGRS+\beta_{2}Duration+\beta_{3}Gender+\beta_{4}UPDRS+\beta_{5}DGRSxDuration+\beta_{6}DGRSxUPDRS)}{{1+exp(\beta }_{0}\left(intercept\right)+\beta_{1}DGRS+\beta_{2}Duration+\beta_{3}Gender+\beta_{4}UPDRS+\beta_{5}DGRSxDuration+\beta_{6}DGRSxUPDRS)}$$

The multivariate binary logistic regression model was validated against a constant model (p = 0.006). The odds of a male having an ICB was more than twice that of a female (odds ratio = 2.26) and significantly contributed to the incidence of an ICB (p = 0.044). As a patient’s UPDRS I&II score increased by 1, they had a 9% increase in the odds of an ICB (β = 0.088, p = 0.01). The incidence of an ICB was over 18 times more likely when a patient had a low compared to medium-range DGRS (β = 2.896, p = 0.04, odds ratio = 18.1). No gene individually showed this association with ICBs (p > 0.357). No other independent variables or interactions increased the likelihood of an ICB.

#### 3.2.2 De novo group

Binary logistic regression model analyses determined the odds of having an ICB increased by 9% with every score increase of 1 on the UPDRS I&II (β = 0.09, p = 0.003, odds ratio = 1.09). Full analyses can be found in supplemental material.

#### 3.2.3 Healthy control group

No independent variables increased the probability of having an ICB in either the univariate or multivariate models (p > 0.382) and the multivariate model was not validated against a constant model (p = 0.761).

#### 3.2.4 QUIP score change onto submit medication

Fig. 1 presents the relationship between QUIP score and time on DA medication for DGRS low, medium and high groups. The number of ICBs increased over time for the high DGRS group but the number of ICBs only tended to decrease over time for medium-range and low DGRS groups. There was a significant positive correlation between QUIP score and days on medication for patients with a high DGRS (r = 0.405, p = 0.033). Correlations between time on medication and QUIP score were negative for patients with a low (r = −0.524, p = 0.120) and medium-range DGRS (r = −0.352, p = 0.152). Fisher Z transformations confirmed a significant difference between correlations in the high and both medium (p = 0.016) and low DGRS groups (p = 0.018), but not between medium-range and low DGRS groups (p = 0.638).

## 4 Discussion

The present study was the first to investigate the relationship between an *a priori*, hypothesis-driven collection of genes and ICBs for people with PD. The novel finding is that dopamine gene profiling via a DGRS has predictive power for ICBs in PD patients on DA medication. As hypothesized, a low DGRS was associated with an increased likelihood of having an ICB on DA medication compared to higher scores. Furthermore, as hypothesized, DGRS influenced the change in ICBs over time on DA medication. Patients with higher DGRS scores increased the number of ICBs with time on DA medication, whereas patients with lower scores tended to reduce ICBs. This association between DGRS and ICBs in the context of an inverted-U relationship between dopamine and impulse control is shown in Fig. 2. The apex of the inverted-U curve signifies the optimal range of dopamine corresponding to maximal impulse control. A reduction in ICBs may reflect a move along the curve towards the apex, and an increase in ICBs a move away from the apex. For all PD patients, regardless of medication status, a higher UPDRS I&II score was associated with increased odds of an ICB. Being male also increased the chance of having an ICB, but only for patients on DA medication. The predictive effects of the DGRS were not present in healthy controls, which supports the contention that the mechanisms of effect are specific to dopamine fluctuations during PD and dopamine therapy.

PD patients’ DGRS were associated with the likelihood of presenting impaired impulse control on DA medication. In the current study, 38% of patients taking DA medication experienced at least one ICB. This percentage aligns with previous reports of 14–40% [30], [3], [4], [5]. Prior studies have found associations between individual dopaminergic gene polymorphisms and ICBs in PD [18], [19], [4], [5], [6]. Of particular note, using the PPMI database Kraemmer et al. [4] reported that neither DRD2, DRD3 nor COMT polymorphisms were individually associated with ICBs. We also found no individual gene was associated with ICBs. It is only by considering the influence of these genes collectively, along with DRD1 as a cumulative polygenic score, that the current study found a significant association (Table 2). This novel finding using a method to quantify the effect of multiple genes simultaneously highlights the importance of considering widespread effects on central dopamine. The resultant association between DGRS and impulse control mirrors that seen in healthy older adults [22]. PD patients with low tonic dopamine levels (i.e. low DGRS) were around 18 times more likely to report an ICB (i.e. worse impulse control) compared to patients with a mid-range DGRS. Impaired impulse control can result from dopamine being either below or above an optimal range, illustrated via the left and right-hand side of the inverted-U curve, respectively (Fig. 2). Low DGRS patients necessarily sit lower on the x axis of this curve, and therefore may present with ICBs as their levels of central dopamine neurotransmission fall below optimal levels.

The DGRS also accounted for changes in impulse control over time on DA medication. Patients with a high DGRS reported worse impulse control with increased time on DA medication, reflected by higher scores on the QUIP. However, patients with both a low and medium DGRS tended to report lower QUIP scores with more time on DA medication. These changes in impulse control over time for all three DGRS groups can also be explained by the inverted-U hypothesis. Greater time on DA medication is illustrated by a rightward shift along the curve (Fig. 2) from increases in neurotransmission. The increase might result from increased medication dosage to combat neurodegenerative disease progression, and/or from decreased sensitivity of D2/D3 autoreceptors [17]. As DA medication dose was not available via the PPMI database we cannot speculate between these potential mechanisms of effect. Either way, an increase in dopamine shifts patients rightwards along the curve, moving patients with a lower DGRS (postulated to sit on the left-hand side) towards optimal levels of dopamine (i.e. the curve apex), but moving high DGRS patients beyond optimal levels. Our results therefore indicate a higher DGRS might be beneficial for impulse control initially, but can be detrimental with exposure to DA medication.

Demographic and clinical factors associated with the presence of ICBs on DA medication replicate previous findings. As hypothesized, male gender and a higher UPDRS I&II score were significantly associated with the presence of an ICB, as previously reported [4], [19], [28]. Sections IV and V of the UPDRS were unavailable, but UPDRS section IV has been found to have associations with ICBs in other studies [19], [28]. Future inclusion of the full UPDRS might reveal interactions with DA medication and/or DGRS..

There are two main limitations of the present study. Firstly, genetic information on the polymorphism within the untranslated region of the DAT gene was unavailable in the PPMI database. The importance of DAT for impulse control behavior and its contribution to the DGRS has been acknowledged [22]. In early PD DAT function is reduced in the MCL system, leading to increased dopamine concentration [10]. With the addition of dopamine medication there can be a dopaminergic overdose within this region, resulting in ICB development [12]. Considering the reduced sensitivity, it is encouraging the DGRS was still able to predict ICB incidence on DA medication without the inclusion of DAT. Nevertheless, it will be beneficial to include DAT within the DGRS in future research. The second limitation is a smaller than desired sample size for the analyses of ICB score. Consequently, we were unable to run a multivariate binary logistic regression model to determine any significant associations between changes in ICB score and clinical, demographic and genetic factors. To investigate these relationships using equivalent multivariate analyses, future studies should use larger cohorts.

In summary, **t**he key finding of the present study was the observed predictive power of a DGRS for ICBs in PD patients on DA medication. An inverted-U relationship between impulse control and dopamine neurotransmission aligns with how DA medication affected patients across the range of DGRS. DA patients with a low DGRS were more likely to have an ICB, but the number of ICBs decreased over time on DA medication. The opposite was observed for the group of patients with a high DGRS, who were less likely to have an ICB on DA medication but over time, the number of ICBs increased. In future research, more sensitive and objective laboratory-based measures could be used in conjunction with a DGRS to identify patients at risk of developing ICBs. This research will help to strengthen the relationship between the utilization of a DGRS and ICB prediction.

## CRediT authorship contribution statement

AH: Data curation, Formal analysis, Writing - original draft. SW: Data curation, Formal analysis, Writing - review & editing. LC: Data curation, Writing - review & editing. WB: Conceptualisation, Writing - review & editing. NJ: Supervision, Writing - review & editing. HM: Conceptualisation, Funding acquisition, Formal analysis, Supervision, Writing - review & editing.

## Declaration of Competing Interest

The authors declare that they have no known competing financial interests or personal relationships that could have appeared to influence the work reported in this paper.
