# Supplementary material for: Dopamine genetic risk score predicts impulse control behaviors in Parkinson’s disease
Source: Clin Park Relat Disord. 2021 Oct 29;5:100113. doi: 10.1016/j.prdoa.2021.100113 (PMC8569744; doi:10.1016/j.prdoa.2021.100113)
Supplement: Supplementary data 1 [file mmc1.docx]

Supplemental material

**DOPAMINE AGONIST (DA) GROUP**

When considering each independent variable in isolation (Table 2S), neither DGRS low (β = 0.313, p = 0.542) or high (β = 0.101, p = 0.791) increased the probability of an ICB in comparison to DGRS medium. This was also the case for duration, where there was no increase in the probability of an ICB with each day (β = -0.01, p = 0.783). However, being male (β = 0.738, p = 0.042) and a higher score on the UPDRS I & II (β = 0.058, p < 0.001) each increased ICB probability. The odds of an ICB in men was 109% higher compared to women, and the probability of an ICB amongst men was 0.68 (p = e^0.738^/1+e^0.738^). When a person’s UPDRS score increased by 1, they had a 6% increase in the odds of an ICB.

**DE NOVO (DN) GROUP**

Of the selected variables (DGRS, gender, UPDRS I&II, age and duration), collinearity was identified between duration and age (p = 0.002), and UPDRS and age (p = 0.023), so age was removed from the model. The relationship between each selected independent variable and the response variable was initially investigated using univariate binary logistic regression analyses.

When considering each independent variable in isolation (Table 3S), only a higher score on the UPDRS I&II (β = 0.047, p = 0.008) increased ICB probability. When a participant’s UPDRS I&II score increased by 1, they had a 5% increase in the odds of an ICB. Having neither a low (β = 0.525, p = 0.266) nor high DGRS (β = -0.178, p = 0.633) had an increased probability of an ICB in comparison to a medium DGRS. Additionally, for each day increase in duration, there was not an increase in the probability of an ICB (β = -0.0003, p = 0.568).

Following univariate analysis (Table 3S), gender was removed from the multivariate model to avoid overparameterization (p = 0.223).

Binary logistic regression function:

$$p =\frac{{exp(\beta}_{0}\left( intercept \right)+ \beta_{1}DGRS + \beta_{2}Duration + \beta_{4}\mathrm{UPDRS}+ \beta_{5}\mathrm{DGRSxDuration}+ \beta_{6}\mathrm{DGRSxUPDRS})}{{1+ exp(\beta}_{0}\left( intercept \right)+ \beta_{1}\mathrm{DGRS} + \beta_{2}\mathrm{Duration} + \beta_{4}\mathrm{UPDRS}+ \beta_{5}\mathrm{DGRSxDuration}+ \beta_{6}\mathrm{DGRSxUPDRS})}$$

The multivariate binary logistic regression model (Table 4S) approached significance when validated against a constant model (p = 0.054). The odds of having an ICB increased by 9% with every score increase of 1 on the UPDRS I&II (β = 0.09, p = 0.003, odds ratio = 1.09). An increase in UPDRS I&II score increased the odds of an ICB in the medium-range DGRS group (odds ratio = e^0.09^ = 1.09) to a greater extent than for those with a high DGRS (odds ratio = e^0.09-0.084^ = 1.01)), although this did not reach significance (p = 0.053). All remaining independent variables and interactions did not change the odds of having an ICB.

To our knowledge this is the first study to investigate genetic associations with ICBs in de novo PD. Current findings show 13% of the de novo group reported an ICB compared to similar studies reporting 17.5% - 18.7%.(1)^,^(2)^,^(3) As expected, this was similar to the 15% reported in the HC group. There was a non-significant trend for increase in ICBs for de novo patients with a low DGRS compared to those with mid-range scores. This trend may be less robust than the relationship found for DA patients due to reduced dopamine disruption in the de novo stage. In the context of the inverted-U hypothesis, less disruption can be conceptualised as a smaller rightward shift for de novo patients compared to DA, resulting in less distinct levels of impulse control between DGRS levels.

UPDRS I&II score was the only factor associated with the incidence of ICBs for de novo patients. Each single point increase in UPDRS I&II score resulted in an increase in the odds of having an ICB. This relationship has not been previously reported in a de novo cohort, only with medicated PD patients.(4)^,^(5) No variable in the current study overlaps with previously reported demographic and clinical factors associated with ICBs in de novo PD, such as being male, a lower Montreal Cognitive Assessment score and a higher Geriatric Depression Scale score.(1)^,^(2)^,^(3) It is clear that a smaller number of factors contribute to ICBs during de novo PD compared to when patients are medicated. This is likely due to reduced dopamine disruption within the MCL system in the de novo stage of PD before DA administration.

**ROC Analyses for DN and DA group**

The ROC curves (Figure 1S) illustrate that the AUC for the DN group multivariate model was 0.62 (95% CI 0.57 to 0.75) for clinical and demographic variables, which increased to 0.66 (95% CI 0.53 to 0.71) with the addition of the DGRS (p = 0.414, DeLong’s test).

The AUC for the DA group was 0.70 (95% confidence interval (CI), upper and lower bounds = 0.61 to 0.78) for clinical and demographic variables, which increased to 0.72 (95% CI 0.64 to 0.81) with the addition of the DGRS (Figure 2S). However, these values were not significantly different (p = 0.326, DeLong’s test).

**Figure legends**

**Figure 1S.** Receiver operating characteristic (ROC) curve for clinical/demographic vs clinical/demographic and genetic associations with incident ICD behaviour (DN group). AUC: area under the curve.

**Figure 2S.** Receiver operating characteristic (ROC) curve for clinical/demographic vs clinical/demographic and genetic associations with incident ICD behaviour (DA group). AUC: area under the curve.

**References:**

1. Antonini A, Siri C, Santangelo G, et al. Impulsivity and Compulsivity in Drug-Naïve Patients with Parkinson’s Disease. *Mov Disord* 2011;26(3):464–8.

2. Weintraub D, Papay K, Siderowf A. Screening for impulse control symptoms in patients with de novo Parkinson disease. *Neurology* 2013;80:176-180.

3. Ryu D-W, Kim J-S, Yoo S-W, et al. The Impact of Impulsivity on Quality of Life in Early Drug-Naïve Parkinson’s Disease Patients. *Mov Disord.* 2019;12(3):172–6.

4. Cormier-Dequaire F, Bekadar S, Anheim M, et al. Suggestive Association Between OPRM1 and Impulse Control Disorders in Parkinson’s Disease. *Mov Disord* 2018;33(12):1878–86.

5. Voon V, Sohr M, Lang AE, et al. Impulse Control Disorders in Parkinson Disease: A multicenter Case-Control Study. *Ann Neurol* 2011;69(6):986–96.
