# Supplementary figures and images for: Dopamine genetic risk score predicts impulse control behaviors in Parkinson’s disease
Source: Clin Park Relat Disord. 2021 Oct 29;5:100113. doi: 10.1016/j.prdoa.2021.100113 (PMC8569744; doi:10.1016/j.prdoa.2021.100113)

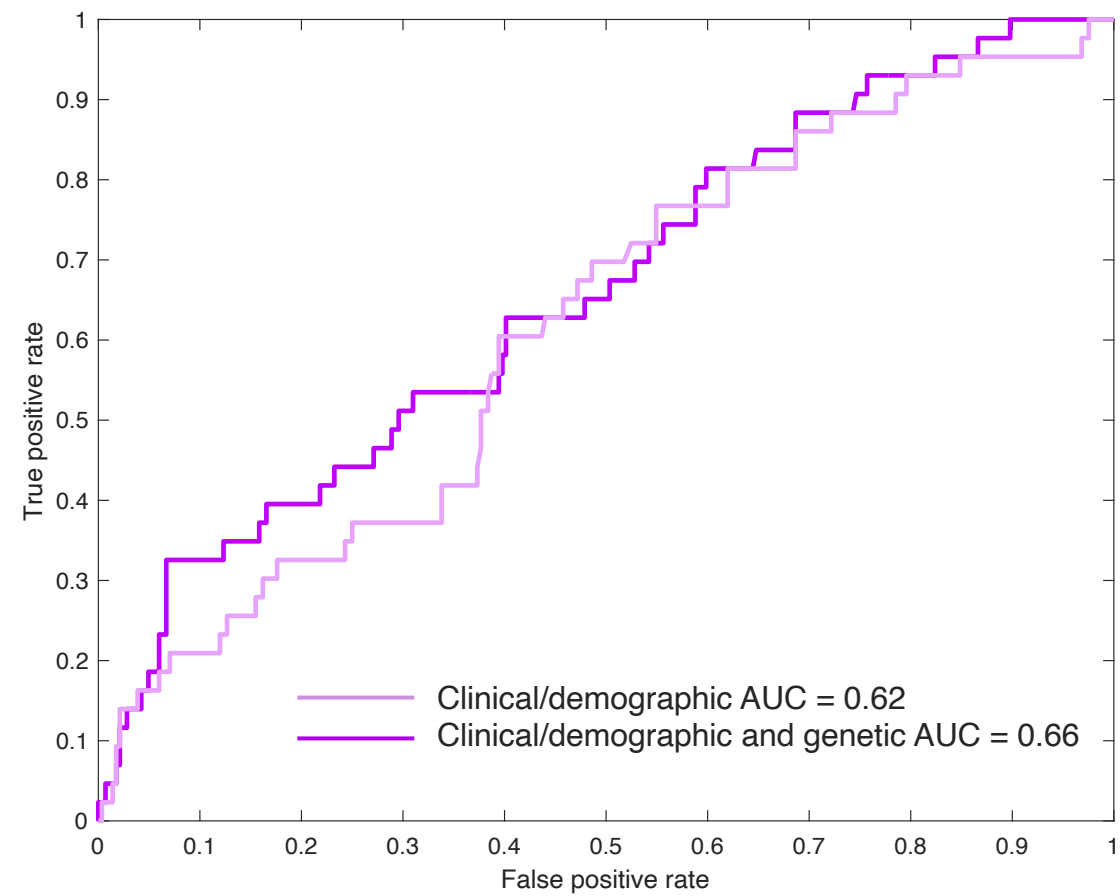

Supplement: Supplementary data 2 [file mmc2.pdf]

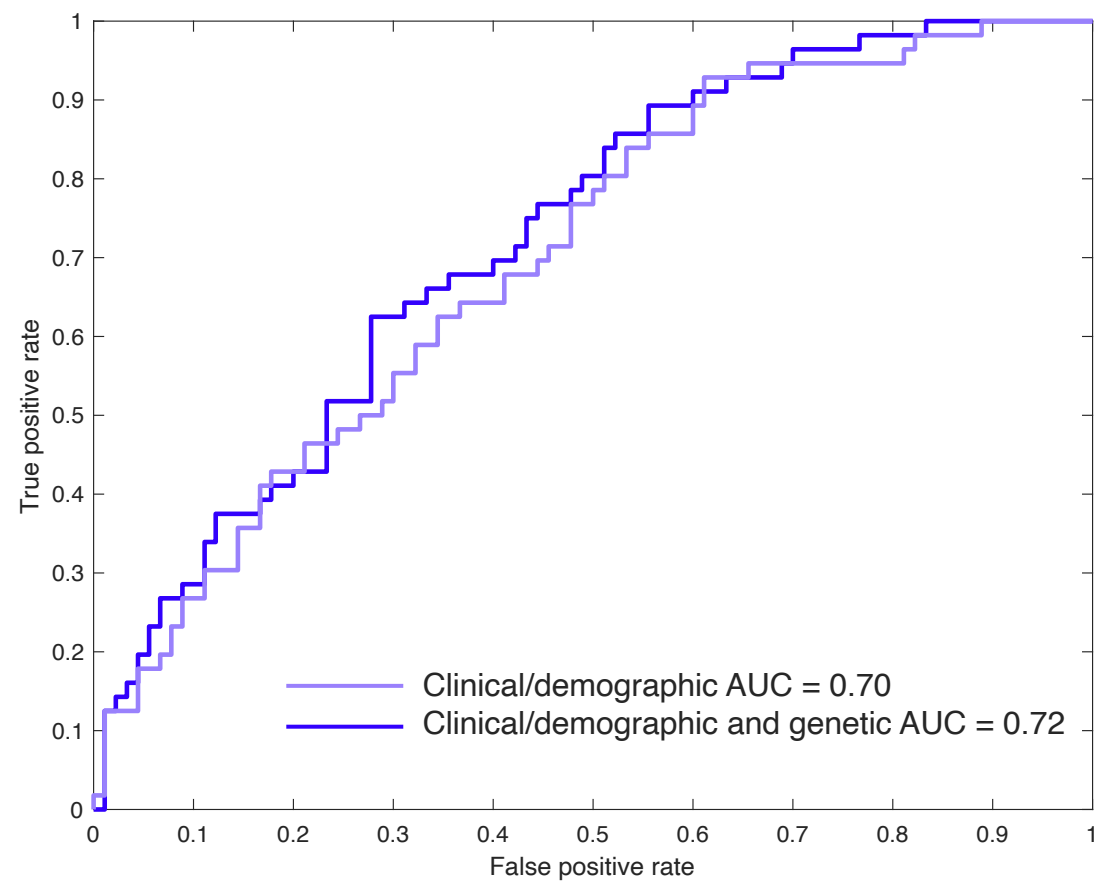

Supplement: Supplementary data 3 [file mmc3.pdf]
