# Supplementary material for: Dopamine genetic risk score predicts impulse control behaviors in Parkinson’s disease
Source: Clin Park Relat Disord. 2021 Oct 29;5:100113. doi: 10.1016/j.prdoa.2021.100113 (PMC8569744; doi:10.1016/j.prdoa.2021.100113)
Supplement: Supplementary data 4 [file mmc4.docx]

|  | DRD1 rs4532 | | | DRD2 rs1800497 | | | DRD3 rs6280 | | | COMT rs4680 | | |
| --- | --- | --- | --- | --- | --- | --- | --- | --- | --- | --- | --- | --- |
|  | A/A | A/G | G/G | C/C | C/T | T/T | T/T | C/T | C/C | G/G | G/A | A/A |
| Score | 0 | 1 | 1 | 1 | 0 | 0 | 0 | 1 | 1 | 0 | 1 | 1 |
| Predict freq | 0.29 | 0.50 | 0.14 | 0.68 | 0.29 | 0.03 | 0.33 | 0.49 | 0.18 | 0.28 | 0.50 | 0.23 |
| Actual freq | 0.42 | 0.45 | 0.13 | 0.67 | 0.27 | 0.06 | 0.52 | 0.38 | 0.10 | 0.32 | 0.39 | 0.29 |

**Table 1S.** Occurrence of polymorphisms for dopamine genetic risk score

﻿DRD1: dopamine receptor D1; DRD2: dopamine receptor D2; DRD3: dopamine receptor D3; COMT: catechol-O-methyltransferase. A: adenine; G: guanine; C: cytosine; T: thymine. Predict freq: expected mutation frequency in population. Actual freq: observed frequency in current population.
