# Supplementary material for: Dopamine genetic risk score predicts impulse control behaviors in Parkinson’s disease
Source: Clin Park Relat Disord. 2021 Oct 29;5:100113. doi: 10.1016/j.prdoa.2021.100113 (PMC8569744; doi:10.1016/j.prdoa.2021.100113)
Supplement: Supplementary data 5 [file mmc5.docx]

**Table 2S.** Variables associated with impulse control behaviours in the dopamine agonist group.

| **Univariate analysis** |  |  |  |  |
| --- | --- | --- | --- | --- |
|  | **β** | **SE** | **p value** | **Odds/OR** |
| DGRS low | 0.313 | 0.514 | 0.542 | 1.37 |
| DGRS high | 0.101 | 0.380 | 0.791 | 1.11 |
| Duration (days) | -0.01 | 0.0003 | 0.783 | 0.99 |
| **Gender (male)** | **0.738** | **0.364** | **0.042** | **2.09** |
| **UPDRS I&II** | **0.058** | **0.017** | **<0.001** | **1.06** |

Response variable: positive score on Questionnaire for Impulsive-Compulsive Disorders in Parkinson’s Disease (yes/no). DGRS: dopamine genetic risk score, UPDRS: Unified Parkinson’s Disease Rating Scale. β: coefficient, SE: standard error, OR: odds ratio (OR = e^β^). Significant values in bold.
