# Supplementary material for: Dopamine genetic risk score predicts impulse control behaviors in Parkinson’s disease
Source: Clin Park Relat Disord. 2021 Oct 29;5:100113. doi: 10.1016/j.prdoa.2021.100113 (PMC8569744; doi:10.1016/j.prdoa.2021.100113)
Supplement: Supplementary data 6 [file mmc6.docx]

**Table 3S.** Variables associated with impulse control behaviours in the de novo group.

| **Univariate analysis** |  |  |  |  |
| --- | --- | --- | --- | --- |
|  | **Coefficient** | **SE** | **p value** | **Odds/OR** |
| DGRS low | 0.525 | 0.471 | 0.266 | 1.69 |
| DGRS high | -0.178 | 0.372 | 0.633 | 0.84 |
| Duration (days) | -0.0003 | 0.0005 | 0.568 | 1.00 |
| Gender (male) | -0.407 | 0.334 | 0.223 | 0.67 |
| **UPDRS I&II** | **0.047** | **0.018** | **0.008** | **1.05** |

Response variable: positive score on Questionnaire for Impulsive-Compulsive Disorders in Parkinson’s Disease (yes/no). DGRS: dopamine genetic risk score, UPDRS: Unified Parkinson’s Disease Rating Scale. β: coefficient, SE: standard error, OR: odds ratio (OR = e^β^). Significant values in bold.
