# Supplementary material for: Dopamine genetic risk score predicts impulse control behaviors in Parkinson’s disease
Source: Clin Park Relat Disord. 2021 Oct 29;5:100113. doi: 10.1016/j.prdoa.2021.100113 (PMC8569744; doi:10.1016/j.prdoa.2021.100113)
Supplement: Supplementary data 7 [file mmc7.docx]

**Table 4S.** Variables associated with impulse control behaviours in the de novo group.

|  | **Coefficient** | **SE** | **p value** | **Odds/OR** |
| --- | --- | --- | --- | --- |
| **Intercept** | **-3.247** | **0.854** | **<0.001** | **0.04** |
| DGRS low | 1.955 | 1.244 | 0.116 | 7.07 |
| DGRS high | 0.947 | 1.045 | 0.365 | 2.58 |
| Duration (days) | -0.0005 | 0.001 | 0.616 | 1.00 |
| **UPDRS I&II** | **0.09** | **0.03** | **0.003** | **1.09** |
| DGRS low * Duration | -0.002 | 0.002 | 0.418 | 1.00 |
| DGRS high * Duration | 0.0008 | 0.001 | 0.511 | 1.00 |
| DGRS low * UPDRS I&II | -0.036 | 0.048 | 0.455 | 0.97 |
| DGRS high * UPDRS I&II | -0.084 | 0.043 | 0.053 | 0.92 |

Response variable: positive score on Questionnaire for Impulsive-Compulsive Disorders in Parkinson’s Disease (yes/no). DGRS: dopamine genetic risk score, UPDRS: Unified Parkinson’s Disease Rating Scale. β: coefficient, SE: standard error, OR: odds ratio (OR = e^β^). Significant values in bold.
